# Joint association of sedentary behavior and physical activity with pulmonary function

**DOI:** 10.1186/s12889-024-18128-2

**Published:** 2024-02-26

**Authors:** Yiwen Wang, Yujie Xie, Yan Chen, Guodong Ding, Yongjun Zhang

**Affiliations:** 1 Xinhua Hospital, Shanghai Jiao Tong University School of Medicine, 1665 Kongjiang Road, Shanghai, 200092 China; 2 Shanghai Institute for Pediatric Research, Xinhua Hospital, Shanghai Jiao Tong University School of Medicine, Shanghai, China

**Keywords:** Sedentary behavior, Moderate or vigorous physical activity, Pulmonary function, NHANES

## Abstract

**Background:**

Sedentary behavior may influence the respiratory health, but the joint effects of sedentary behavior and physical activity on pulmonary function remains poorly elucidated. We aimed to estimate the association between sedentary behavior and physical activity with pulmonary function.

**Methods:**

A total of 12,343 participants aged 12–79 years were analyzed from the U.S. NHANES 2007–2012. Participants were categorized into 16 groups according to the cross-tabulation of sedentary behavior time (0–4.0, 4.1–8.0, 8.1–12.0, and > 12.0 h/day) and moderate or vigorous physical activity (MVPA) (0, 1–149, 150–299, and ≥ 300 min/week). Generalized linear models were used to test the association of sedentary behavior and MVPA with pulmonary function.

**Results:**

Participants with sedentary behavior > 4.0 h/day were negatively related to FEV_1_ (forced expiratory volume in 1 s) (β ranging from -0.015 to -0.009, *p* < 0.05). Compared with the reference group (0 min of MVPA and > 12.0 h/day of sedentary behavior), the negative association of sedentary behavior ≤ 8.0 h/day with FEV_1_ may be reduced through appropriate MVPA (β ranging from 0.019 to 0.030, *p* < 0.05). For sedentary behavior > 8.0 h/day, even MVPA ≥ 300 min/week may not decrease the negative relationships. Similar results were also observed in FVC (forced vital capacity) (β ranging from 0.018 to 0.030, *p* < 0.05). In participants aged ≥ 45 years, the associations were more notable.

**Conclusion:**

This study indicated the sedentary behavior ≤ 4.0 h/day was a relatively healthy lifestyle for pulmonary function. Only below 8.0 h/day of sedentary behavior, the negative association with pulmonary function may be reduced through appropriate MVPA.

**Supplementary Information:**

The online version contains supplementary material available at 10.1186/s12889-024-18128-2.

## Background

Sedentary behavior has been involved in lower health-related quality of life, higher rates of mood disorders and depressive symptoms, and higher prevalence of chronic illnesses [1–3]. Although the association between sedentary behavior and pulmonary function has been elucidated [4–6], few have so far identified a critical threshold value for the relationship of sedentary time with pulmonary function [7].

National and global guidelines on physical activity are a core ingredient of a coherent and comprehensive policy and governance framework for public health action. Physical activity has been identified to benefit populations with chronic diseases, including heart disease, high blood pressure, diabetes, and impaired pulmonary function [8–10]. It was reported that daily being 1 h less physical activity was related to a lower forced expiratory volume in 1 s (FEV_1_)/forced vital capacity (FVC) in childhood [10]. In the real world, however, prolonged sedentary behavior is sometimes unavoidable and the situation is further complicated by the fact that prolonged sedentary behavior and insufficient physical activity may have some effect on each other, modifying the relationship with some health outcomes and working together on public health [11]. It was reported that the association of sedentary behavior with all-cause mortality or cardiovascular disease mortality depended on MVPA levels [12, 13], in which high levels of moderate intensity physical activity appeared to eliminate the increased risk of death associated with high sedentary behavior time [14, 15]. This implies that it is more valuable to explore the joint association of sedentary behavior and physical activity with pulmonary function than either of them alone. In this study, we hypothesized that the negative association of longer sedentary behavior could be reduced to some degree by appropriate extension of physical activity time. Therefore, we aimed to investigate the joint relationships of sedentary behavior and physical activity with pulmonary function and quantify the amount of physical activity required to reduce the negative associations of various sedentary behaviors and pulmonary function, which may help develop practical guidelines for population health.

## Methods

### Study setting and design

The U.S. National Health and Nutrition Examination Study (NHANES) is a series of nationally representative cross–sectional studies. The National Center for Health Statistics (NCHS) sponsored and approved NHANES [16]. Detailed descriptions and protocols about the NHANES study can be found online at http://www.cdc.gov/nchs/nhanes.htm. All participants provided written informed consent, and all procedures were approved by the Institutional Ethics Review Board of NCHS. Because the data are publicly available and anonymized, the Ethical Committee and Institutional Review Board Committee of Xinhua Hospital considered the study exempt from ethics review. The investigation conforms to the principles outlined in the Declaration of Helsinki.

**Fig. 1:**
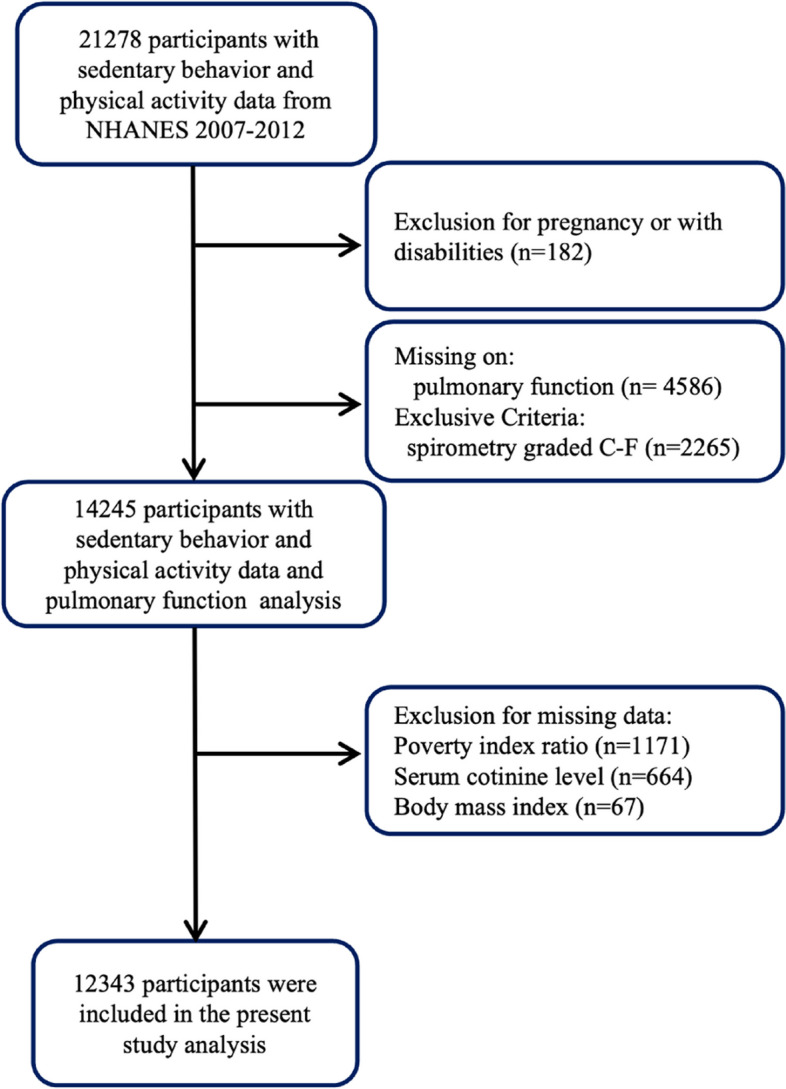
Flow chart

Data from participants recruited from 2007 to 2012 were used. A total of 21,278 individuals aged 12–80 years, including four age groups, had information about sedentary behavior and physical activity. We excluded individuals who were pregnant or had a history of disabilities (*n* = 182). Individuals with missing data on measures of spirometry (*n* = 4586) and spirometry graded C-F (*n* = 2265) were excluded. We further excluded individuals whose data on covariates, including body mass index (BMI), poverty index ratio, or serum cotinine level, were missing (*n* = 1902), leaving 12,343 individuals with complete information about covariates for the final analysis (Fig. 1).

### Assessment of sedentary behavior and physical activity

Data on sedentary behavior and physical activity was self-reported in NHANES using the Global Physical Activity Questionnaire developed by the World Health Organization [17], which has been extensively applied worldwide with robust evidence of validity and reliability across diverse population groups [18]. Sedentary behavior is defined as activities that do not increase energy expenditure above the resting level and includes time spent on activities such as watching TV, working on a computer, lying down and sitting during waking hours, and engaging in other forms of screen-based entertainment [19]. The duration of sedentary behavior was calculated using the self-reported time usually spent sitting on a typical day (PAD 680) [20]. Sedentary behavior time was categorized into four groups (0–4.0, 4.1–8.0, 8.1–12.0, and > 12.0 h/day) [21]. Participants were inquired about the average number of days and duration spent on recreational physical activity daily volume for ≥ 10 min at a time. According to the World Health Organization Guidelines on sedentary behavior and physical activity [22], individuals who engaged in ≥ 150 min/week of moderate-intensity aerobic physical activity, ≥ 75 min/week of vigorous-intensity aerobic physical activity, or had an equivalent combination of moderate and vigorous physical activity (1 min of vigorous-intensity physical activity is equivalent to 2 min of moderate-intensity physical activity) totaling at least 150 min/week were defined as meeting the guidelines. According to the reported number of days and time in minutes spent on moderate or vigorous recreational physical activity (MVPA), individuals were classified into four ordinal groups: no physical activity, 1–149, 150–299, and 300 min/week or more [23].

### Outcome assessment

Participants taking medication for tuberculosis, with persistent cough, with an acute respiratory condition, and those who had recent abdominal or thoracic surgery were excluded from pulmonary function testing. The spirometry testing protocol and quality control procedures followed the American Thoracic Society (ATS) guidelines [24]. Each subject performed the test at most eight times to achieve at least three reproducible and acceptable criteria. The FEV_1_ and FVC were rated by quality grade (A to F), and we excluded individuals with FVC and FEV_1_ values grade C-F [25]. Grade A = Exceeds ATS data collection standards: 3 acceptable curves present and 2 reproducible curves; 2 observed values within 100 ml. Grade B = Meets ATS data collection standards: 3 acceptable curves present and 2 reproducible curves; 2 observed values within 150 ml. Grade C = Potentially usable value, but does not meet all ATS standards. Estimates are usually based on two curve results with values within 200 ml of each other. Grade D = Questionable result, use with caution. Grade F = Results not valid. We focused our data analysis on five pulmonary function metrics, which included FEV_1_, FVC, ratio of FEV_1_ to FVC (FEV_1_:FVC), peak expiratory flow (PEF), and forced expiratory flow between 25 and 75% of vital capacity (FEF_25-75%_).

### Sociodemographic covariates

Trained interviewers administered questionnaires to individuals and collected demographic information on poverty status, sex, race/ethnicity, and age. Race/ethnicity was categorized into “Mexican American”, “Non-Hispanic Black”, “Non-Hispanic White”, “Other Hispanic” and “Other Race-including multi-Racial”. Serum cotinine levels were measured and classified as ≥ LOD (limits of detection, 0.015 ng/mL), and < LOD, which could reflect the exposure status of environmental tobacco smoke [26]. The poverty income ratio was calculated by dividing the household income by the poverty guidelines of a specific survey year [27]. BMI was calculated as the weight in kilograms divided by the square height in meters (kg/m^2^). Lung disease was defined by answers to the questions, “Have you had wheezing or whistling in your chest in the past one year?”, “Has a doctor or other health professional ever told you that you have chronic bronchitis?”, “Has a doctor or other health professional ever told you that you have emphysema?”, and “Has a doctor or other health professional ever told you that you have asthma?”.

## Statistical analysis

Descriptive statistical analyses were calculated to summarize the demographic characteristics, sedentary behavior, physical activity, and pulmonary function measures. The spirometric measurements (FVC, FEV_1_, FEV_1_: FVC, PEF, and FEF_25-75%_) were log_10_-transformed and handled as continuous variables in the models. Tests for trend (p-trend) were conducted using median sedentary behavior time in each ordinal category as a continuous variable. Participants were then categorized into 16 groups according to the cross-tabulation of sedentary behavior time (0–4.0, 4.1–8.0, 8.1–12.0, and > 12.0 h/day) and MVPA (0, 1–149, 150–299, and 300 min/week or more), and the joint analyses were conducted by generalized linear models using the group of sedentary behavior > 12.0 h/day and 0 min of MVPA as the reference group. Stratified analysis was conducted to explore potential effect modifications by sex and age groups (12–19, 20–44, 45–59, and 60–79 years). We also did sensitivity analyses restricted to individuals without lung diseases. Potential confounders were selected from previous studies of respiratory conditions, including poverty income ratio, serum cotinine levels, NHANES cycles, age, BMI, race, height, sex, and chronic lung diseases [7, 27]. All analyses were conducted with adjustments for complex, multistage sampling survey designs (e.g., stratification and clusters) and were conducted using SAS version 9.4 (SAS Institute Inc, Cary, NC) and OriginPro Version 2021 (Origin Lab Corporation, Northampton, MA, USA.) *P* < 0.05 (two-tailed) was considered statistically significant.

## Results

Table 1 presents the socio-demographic characteristics of 12,343 participants. About 41.4% of the participants had a sedentary behavior ≤ 4.0 h/day, 37.7% for 4.1–8.0 h/day, 17.9% for 8.1–12.0 h/day, and 3.0% for > 12.0 h/day. There are 2316 participants at the age of 12–19 years (18.8%), 4813 at 20–44 years (39.0%), 2695 at 45–59 years (21.8%), and 2519 at 60–79 years (20.4%). Approximately half (50.3%) of the participants were males. The median (95% CI) for FVC, FEV_1_, FEV_1_: FVC, PEF, and FEF_25-75%_ were 4043.9 (4011.0, 4076.8) mL, 3217.1 (3191.8, 3242.4) mL, 0.80 (0.80, 0.80), 8107.6 (8016.4, 8198.8) mL/s, and 3088.2 (3031.6, 3144.7) mL/s, respectively (Supplementary Table 1).

**Table 1:** Characteristics of the study population (*n* = 12,343)

Characteristics	**Total**	**Sedentary behavior time**				**MVPA time**			
	**Mean ± SD or n (%)**	**0–4.0 h/day**	**4.1–8.0 h/day**	**8.1–12.0 h/day**	**> 12.0 h/day**	**0 min/week**	**1–149 min/week**	**150–299 min/week**	≥ **300 min/week**
		*n* = 5113 (41.4%)	*n* = 4657 (37.7%)	*n* = 2207 (17.9%)	*n* = 366 (3.0%)	*n* = 5391 (43.7%)	*n* = 1848 (15.0%)	*n* = 1495 (12.1%)	*n* = 3609 (29.2%)
**Sex**									
Male	6205 (50.3)	2685 (52.5)	2269 (48.7)	1062 (48.1)	189 (51.6)	2454 (45.5)	808 (43.7)	719 (48.1)	2224 (61.6)
Female	6138 (49.7)	2428 (47.5)	2388 (51.3)	1145 (51.9)	177 (48.4)	2937 (54.5)	1040 (56.3)	776 (51.9)	1385 (38.4)
**Age (years)**									
12–19 years	2316 (18.8)	421 (8.2)	1058 (22.7)	754 (34.2)	83 (22.7)	525 (9.7)	272 (14.7)	271 (18.1)	1248 (34.6)
20–44 years	4813 (39.0)	2212 (43.3)	1722 (37.0)	735 (33.3)	144 (39.3)	1983 (36.8)	771 (41.7)	601 (40.2)	1458 (40.4)
45–59 years	2695 (21.8)	1283 (25.1)	916 (19.7)	413 (18.7)	83 (22.7)	1458 (27.0)	407 (22.0)	319 (21.3)	511 (14.2)
≥ 60 years	2519 (20.4)	1197 (23.4)	961 (20.6)	305 (13.8)	56 (15.3)	1425 (26.4)	398 (21.5)	304 (20.3)	392 (10.9)
**BMI (kg/m**^**2**^**)**	28.20 (6.89)	28.26 (6.18)	28.19 (7.09)	27.91 (7.76)	29.13 (8.09)	29.57 (7.40)	28.37 (6.77)	27.50 (6.31)	26.35(5.93)
**Race/ethnicity**									
Non–Hispanic White	5355 (43.4)	2051 (40.1)	2112 (45.4)	1011 (45.8)	181 (49.5)	2178 (40.4)	876 (47.4)	722 (48.3)	1579 (43.8)
Non–Hispanic Black	2536 (20.5)	977 (19.1)	1008 (21.6)	466 (21.1)	85 (23.2)	1166 (21.6)	348 (18.8)	257 (17.2)	765 (21.2)
Mexican American	2103 (17.0)	1134 (22.2)	661 (14.2)	272 (12.3)	36 (9.8)	1028 (19.1)	263 (14.2)	249 (16.7)	563 (15.6)
Other Hispanic	1305 (10.6)	630 (12.3)	457 (9.8)	204 (9.2)	14 (3.8)	682 (12.7)	173 (9.4)	118 (7.9)	332 (9.2)
Other Race-including Multi-Racial	1044 (8.5)	321 (6.3)	419 (9.0)	254 (11.5)	50 (13.7)	337 (6.3)	188 (10.2)	149 (10.0)	370 (10.3)
**Poverty index ratio**									
Below poverty level (≤ 1)	2806 (22.7)	1276 (25.0)	1066 (22.9)	409 (18.5)	55 (15.0)	1429 (26.5)	355 (19.2)	267 (17.9)	755 (20.9)
Above poverty level (> 1)	9537 (77.3)	3837 (75.0)	3591 (77.1)	1798 (81.5)	311 (85.0)	3962 (73.5)	1493 (80.8)	1228 (82.1)	2854 (79.1)
**Serum cotinine level**									
< LOD	2729 (22.1)	995 (19.5)	1066 (22.9)	579 (26.2)	277 (75.7)	1005 (18.6)	462 (25.0)	396 (26.5)	866 (24.0)
≥ LOD	9614 (77.9)	4118 (80.5)	3591 (77.1)	1628 (73.8)	89 (24.3)	4386 (81.4)	1386 (75.0)	1099 (73.5)	2743 (76.0)
**NHANES cycles**									
2007–2008	3970 (32.2)	1902 (37.2)	1335 (28.7)	624 (28.3)	109 (29.8)	1844 (34.2)	564 (30.5)	441 (29.5)	1121 (31.1)
2009–2010	4423 (35.8)	1859 (36.4)	1722 (37.0)	727 (32.9)	115 (31.4)	1957 (36.3)	661 (35.8)	556 (37.2)	1249 (34.6)
2011–2012	3950 (32.0)	1352 (26.4)	1600 (34.4)	856 (38.8)	142 (38.8)	1590 (29.5)	623 (33.7)	498 (33.3)	1239 (34.3)
**Chronic lung disease and/or Chronic bronchitis-like symptoms**^a^									
Yes	2913 (23.6)	1126 (22.0)	1112 (23.9)	566 (25.6)	109 (29.8)	1352 (25.1)	441 (23.9)	304 (20.3)	816 (22.6)
No	9430 (76.4)	3987 (78.0)	3545 (76.1)	1641 (74.4)	257 (70.2)	4039 (74.9)	1407 (76.1)	1191 (79.7)	2793 (77.4)

*SD* Standard deviation, *MVPA* Moderate or vigorous physical activity, *BMI* Body mass index, *LOD* Limit of detection

^a^Lung disease was defined by answers to the questions “Has a doctor or other health professional ever told you that you have asthma?”, “Has a doctor or other health professional ever told you that you have emphysema?”, “Has a doctor or other health professional ever told you that you have chronic bronchitis?” and “Have you had wheezing or whistling in your chest in the past one year?”

### Individual or joint effects of sedentary behavior and MVPA on pulmonary function

Excepted FEV_1_:FVC, sedentary behavior as a categorical variable was negatively related to the other four spirometry (*p*-trend < 0.05) (Supplementary Table 2). Compared with the reference group (0–4.0 h/day of sedentary behavior), participants with sedentary behavior > 4.0 h/day was negatively related with FEV_1_ (β: -0.009, 95%CI: -0.014– -0.004 for sedentary behavior of 4.1–8.0 h/day; β: -0.015, 95%CI: -0.020– -0.009 for sedentary behavior of 8.1–12.0 h/day; and β: -0.015, 95%CI: -0.026– -0.004 for sedentary behavior > 12.0 h/day) and FVC (β: -0.011, 95%CI: -0.015– -0.008 for sedentary behavior of 4.1–8.0 h/day; β: -0.015, 95%CI: -0.019– -0.010 for sedentary behavior of 8.1–12.0 h/day; and β: -0.016, 95%CI: -0.026– -0.006 for sedentary behavior > 12.0 h/day).

**Fig. 2:**
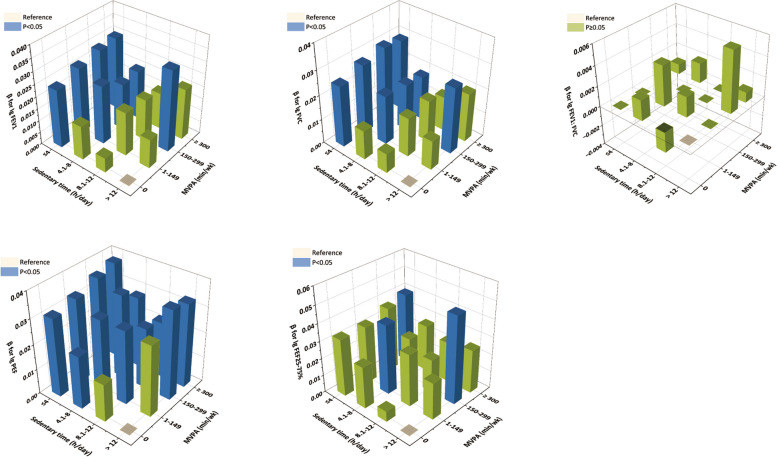
Joint association between sedentary behavior, MVPA time and pulmonary function in the NHANES 2007–2012. The model was adjusted for sex, age, family income to poverty ratio, height, body mass index, race, serum cotinine, lung diseases, and NHANES cycles. FEV_1_: forced expiratory volume in 1 s; FVC: forced vital capacity; PEF: peak expiratory flow; FEF_25-75%_: forced expiratory flow between 25 and 75% of vital capacity; MVPA: moderate or vigorous physical activity

In the joint sedentary-MVPA models (Table 2, Fig. 2), compared with the reference group (0 min of MVPA and > 12.0 h/day of sedentary behavior), participants with sedentary behavior ≤ 4.0 h/day have significantly enhanced FEV_1_, which rises with increasing MVPA time (β ranging from 0.024 to 0.030, *p* < 0.05). In those with sedentary behavior of 4.1–8.0 h/day, MVPA also has a significant association with FEV_1_, but to milder extents (β ranging from 0.019 to 0.024, *p* < 0.05), and a total of up to 150 min/week MVPA may be enough for these participants, as there does not appear to be any further significant increase of FEV_1_ beyond 150 min/week MVPA. For those with sedentary behavior > 8.0 h/day, even MVPA ≥ 300 min/week could not reduce the negative associations of sedentary behavior and FEV_1_. Similar results were observed in the sedentary-MVPA model with FVC as the outcome. Also, for PEF, MVPA has a positive role in participants with sedentary behavior ≤ 8.0 h/day regardless of the amount of MVPA time (β ranging from 0.021 to 0.038, *p* < 0.05) and the shorter the sedentary behavior time, the higher PEF is achieved. While in those with sedentary behavior > 8.0 h/day, more MVPA is needed.

**Table 2:** Joint association of sedentary behavior, MVPA time and pulmonary function in the NHANES 2007–2012

	FEV_1_	FVC	FEV_1_: FVC	PEF	FEF_25-75%_
	β (95%CI)^a^				
**> 12 h/day SB and 0 min/wk MVPA**	**Reference**	**Reference**	**Reference**	**Reference**	**Reference**
**> 12 h/day SB and 1–149 min/wk MVPA**	0.012 (-0.013, 0.037)	0.012 (-0.011, 0.035)	0.000 (-0.011, 0.011)	0.028 (-0.003, 0.058)	0.021 (-0.031, 0.072)
**> 12 h/day SB and 150–299 min/wk MVPA**	0.033 (0.012, 0.053)^*^	0.027 (0.005, 0.048)^*^	0.006 (-0.003, 0.016)	0.035 (0.010, 0.059)^*^	0.050 (0.004, 0.096)^*^
**> 12 h/day SB and ≥ 300 min/wk MVPA**	0.021 (-0.003, 0.044)	0.020 (-0.002, 0.042)	0.001 (-0.010, 0.011)	0.033 (0.010, 0.057)^*^	0.025 (-0.022, 0.073)
**8.1–12 h/day SB and 0 min/wk MVPA**	0.006 (-0.012, 0.024)	0.008 (-0.009, 0.026)	-0.002 (-0.011, 0.007)	0.015 (-0.003, 0.033)	0.006 (-0.032, 0.044)
**8.1–12 h/day SB and 1–149 min/wk MVPA**	0.018 (-0.002, 0.038)	0.016 (-0.003, 0.035)	0.002 (-0.006, 0.010)	0.029 (0.011, 0.048)^*^	0.030 (-0.008, 0.069)
**8.1–12 h/day SB and 150–299 min/wk MVPA**	0.017 (-0.002, 0.036)	0.017 (-0.000, 0.035)	0.000 (-0.009, 0.009)	0.024 (0.006, 0.043)^*^	0.019 (-0.023, 0.061)
**8.1–12 h/day SB and ≥ 300 min/wk MVPA**	0.015 (-0.002, 0.031)	0.014 (-0.001, 0.029)	0.000 (-0.007, 0.007)	0.022 (0.006, 0.039)^*^	0.023 (-0.014, 0.060)
**4.1–8 h/day SB and 0 min/wk MVPA**	0.014 (-0.006, 0.033)	0.012 (-0.005, 0.030)	0.002 (-0.005, 0.009)	0.021 (0.003, 0.039)^*^	0.024 (-0.014, 0.062)
**4.1–8 h/day SB and 1–149 min/wk MVPA**	0.024 (0.004, 0.044)^*^	0.020 (0.001, 0.039)^*^	0.004 (-0.004, 0.011)	0.029 (0.013, 0.046)^*^	0.040 (0.004, 0.077)^*^
**4.1–8 h/day SB and 150–299 min/wk MVPA**	0.019 (0.000, 0.038)^*^	0.019 (0.001, 0.036)^*^	0.000 (-0.007, 0.008)	0.033 (0.016, 0.050)^*^	0.025 (-0.013, 0.064)
**4.1–8 h/day SB and ≥ 300 min/wk MVPA**	0.020 (0.002, 0.038)^*^	0.018 (0.001, 0.035)^*^	0.002 (-0.005, 0.010)	0.028 (0.011, 0.045)^*^	0.027 (-0.007, 0.061)
**0–4 h/day SB and 0 min/wk MVPA**	0.024 (0.005, 0.043)^*^	0.025 (0.007, 0.042)^*^	0.000 (-0.008, 0.008)	0.031 (0.014, 0.047)^*^	0.033 (-0.004, 0.070)
**0–4 h/day SB and 1–149 min/wk MVPA**	0.027 (0.009, 0.046)^*^	0.028 (0.011, 0.046)^*^	0.000 (-0.008, 0.008)	0.033 (0.015, 0.052)^*^	0.033 (-0.005, 0.071)
**0–4 h/day SB and 150–299 min/wk MVPA**	0.029 (0.008, 0.051)^*^	0.030 (0.011, 0.049)^*^	0.000 (-0.009, 0.009)	0.036 (0.016, 0.055)^*^	0.037 (-0.006, 0.080)
**0–4 h/day SB and ≥ 300 min/wk MVPA**	0.030 (0.012, 0.048)^*^	0.029 (0.012, 0.045)^*^	0.001 (-0.006, 0.009)	0.038 (0.021, 0.055)^*^	0.040 (0.003, 0.077)^*^

FEV_1_, FVC, FEV_1_: FVC, PEF, and FEF_25-75%_ were log_10_-transformed

*FEV*_*1*_ Forced expiratory volume in 1 s, *FVC* Forced vital capacity, *PEF* Peak expiratory flow, *FEF*_*25-75%*_ Forced expiratory flow between 25 and 75% of vital capacity, *MVPA* Moderate or vigorous physical activity, *SB* Sedentary behavior

^*^Means *P* < 0.05

^a^Adjusted for sex, age, family income to poverty ratio, height, BMI (body mass index), race, serum cotinine, lung diseases, and NHANES cycles

Stratified analyses by age and sex were performed to estimate the joint association of sedentary behavior and MVPA with pulmonary function respectively. Similar results are observed between males and females (Supplementary Table 3, Supplementary Figs. 1 and 2). With regard to age, in participants aged ≥ 45 years, the positive associations were more notable (Supplementary Table 4, Supplementary Figs. 3, 4, 5 and 6). Specifically, in participants aged 45–59 years with sedentary behavior ≤ 4.0 h/day, any amount of MVPA may be involved in elevated FEV_1_ and FVC (β ranging from 0.036 to 0.043 for FEV_1_, and 0.034 to 0.037 for FVC). While in those with sedentary time > 4.0 h/day, only MVPA ≥ 300 min/week has a positive relationship with FEV_1_ (β: 0.046, 95%CI: 0.004–0.087 for sedentary behavior > 12.0 h/day; β: 0.045, 95%CI: 0.014–0.076 for sedentary behavior of 8.1–12.0 h/day; β: 0.044, 95%CI: 0.014–0.073 for sedentary behavior of 4.1–8.0 h/day) and FVC (β: 0.035, 95%CI: 0.009–0.062 for sedentary behavior of 8.1–12.0 h/day; β: 0.032, 95%CI: 0.008–0.056 for sedentary behavior of 4.1–8.0 h/day). For PEF, compared to reference groups, MVPA almost plays positive roles in all groups, and PEF reaches the highest level at MAPA ≥ 300 min/week (β ranging from 0.032 to 0.060, *p* < 0.05). Meanwhile, in participants aged ≥ 60 years with sedentary behavior ≤ 8.0 h/day, any amount of MVPA may improve FEF_25-75%_ (β ranging from 0.076 to 0.135, *p* < 0.05), but not for FEV_1_, FVC, or PEF. No effect of MVPA on FEV_1_:FVC is presented.

### Sensitivity analysis and data imputation

In the sensitivity analyses, when participants were restricted to individuals without lung disease, the relationship of sedentary behavior and MVPA with PEF was similar, and it also persisted in those with sedentary behavior ≤ 4.0 h/day when FEV_1_ and FVC were considered as outcomes (Supplementary Table 5, Supplementary Fig. 7). As 1902 participants whose information on covariates, including BMI, poverty index ratio, or serum cotinine level, were missing, PROC MI procedure and PROC MIANALYZE were used for data imputation and the joint association of sedentary behavior and MVPA with pulmonary function also remained similar (Supplementary Table 6, Supplementary Fig. 8).

## Discussion

For pulmonary function, we demonstrated that sedentary ≤ 4.0 h/day is the threshold, and the negative associations with pulmonary function may be partly attenuated by appropriate MVPA. For specific participants aged 45–59 years with sedentary behavior ≤ 4.0 h/day, any amount of MVPA may be helpful. For those with sedentary behavior > 4.0 h/day, the recommended MVPA may be 300 min/week and above. In participants over 60 years of age, MVPA may attenuate the negative association between sedentary behavior ≤ 8.0 h/day and FEF_25-75%_.

### Comparison with other studies

Prolonged sedentary behavior was one of the risk factors for a variety of chronic diseases, such as obesity and metabolic syndrome related to metabolic disturbances [28], all-cause and cardiovascular disease mortality [15], cardiovascular diseases [29], and pulmonary function [4–6]. For researches on pulmonary function, although Mensink-Bout et al. reported a higher screen time was associated with a lower FVC [30], the sedentary behavior threshold remained unclear. In the present study, we found that the degree of improvement in pulmonary function by MVPA may be dependent on sedentary time to some extent, the shorter the sedentary time, the more improvement by MVPA. The sedentary behavior ≤ 4.0 h/day was a relatively healthy lifestyle, in which spirometric measurements rose with increasing MVPA time. In terms of sedentary behavior ≤ 8.0 h/day, the negative association could be reduced by appropriate MVPA. In contrast, it could not be eliminated despite MVPA ≥ 300 min/week, in cases of sedentary behavior > 8.0 h/day. Of note, in those without lung disease, individuals with longer sedentary time (> 4.0 h/day) did not appear to be sensitive to MVPA, implying that it may still be necessary for them to reduce sedentary time to prevent the negative effects on spirometric measurements. Somewhat different from our focus on the relationship of MVPA duration to pulmonary function, previous studies on different types of physical activity confirmed that any type may also bring about benefits to pulmonary function [7]. These can potentially be attributed to the fact that prolonged sedentary behavior may lead to physiological changes that accelerate age-related declines in pulmonary function and increase the risk of developing respiratory diseases [7, 31–33].

Our suggestions for MVPA are grading by groups of sedentary behavior time and age. The WHO guideline recommends that adults should engage in 150–300 min/week of moderate-intensity, or 75–150 min/week of vigorous-intensity aerobic physical activity, or some equivalent combinations of both [22]. In our study, we give specific suggestions for participants aged 45–59 years and 60–79 years, respectively. In participants aged 45–59 years with sedentary behavior ≤ 4.0 h/day, any amount of MVPA may be helpful; while for those with sedentary behavior > 4.0 h/day, MVPA may be 300 min/week and above. As for participant over 60 years of age, MVPA may eliminate the negative relationship between sedentary behavior ≤ 8.0 h/day and FEF_25-75%_. In the present study, we demonstrated the threshold of sedentary behavior on pulmonary function, and the negative associations of sedentary behavior and pulmonary function may be partly attenuated by appropriate MVPA, Furthermore, we made customized suggestion, not only for various sedentary behavior time but also for various age groups. These evidence might underpin the guidance on physical activity and sedentary behavior in the future.

### Potential mechanisms

The role of MVPA in attenuating the negative associations of sedentary behavior and pulmonary function may be owning to the positive effect on inspiratory muscle endurance, or enhancing expiratory muscle contraction strength, or counteracting impact that physical activity may have on the age related chest wall stiffening, or a smooth muscle relaxation-induced modulation of resistance to airflow and airway diameter [34–36]. The mechanisms are likely to be associated with an increased sympathetic activity during physical activity, which subsequently trigger a decrement of airway resistance through ß2 receptor activation-induced reduced airway smooth muscle tone [36]. Furthermore, it has been reported that the increased sympathetic actiity during physical activity can also lead to an enhanced expiratory muscle strength, which is an important factor for improving pulmonary function [37–39].

### Strengths and limitations of the study

The present study has two strengths. Firstly, NHANES is a large study that collects data using a standardized study protocol, employs extensive quality control measures, and utilizes technicians who are certified in data collection procedures. Secondly, instead of the individual risk factor, we explore the joint association of sedentary behavior and physical activity with pulmonary function to imitate the real world. Nevertheless, this study also has several limitations. Firstly, information on sedentary behavior and MVPA was collected from the questionnaires and may be subject to recall bias. Secondly, since it was an observational cross-sectional study, the causal association of sedentary behavior and MVPA with pulmonary function cannot be inferred. Thirdly, the sample is just from the USA, which limits the generalization of the results to other countries with different populations, socioeconomic, demographic, and cultural characteristics. Lastly, in the present study, the conclusions deduced from the present study were based on the outcomes of spirometric test. Thus, clinicians should be prudent when making extrapolation and need to comprehensively consider the relevant recommendations from WHO and the associations between MVPA and other conditions.

## Conclusion

This study indicated that a higher sedentary behavior time was associated with lower pulmonary function, and sedentary behavior ≤ 4.0 h/day was a relatively healthy lifestyle for pulmonary function. The negative association between sedentary behavior and pulmonary function may be reduced through appropriate MVPA to some extent. Clinicians and public health interventions encourage populations to decrease time spent on sedentary behavior as far as possible, since the negative association of sedentary time and pulmonary function might not be eliminated despite increased physical activity, in cases of too long sedentary behavior time.

### Supplementary Information

**Supplementary Material 1. **

## Data Availability

Data that support the findings of this study are available from the corresponding authors upon a reasonable request.

## Abbreviations

FEV_1_: Forced expiratory volume in 1 s
FVC: Forced vital capacity
NHANES: National Health and Nutrition Examination Survey
NCHS: National Center for Health Statistics
BMI: Body mass index
MVPA: Moderate or vigorous physical activity
LOD: Limits of detection
PEF: Peak expiratory flow
FEF_25-75%_: Forced expiratory flow between 25 and 75% of vital capacity
CI: Confidence interval
