# Supplementary material for: Joint association of sedentary behavior and physical activity with pulmonary function
Source: BMC Public Health. 2024 Feb 26;24:604. doi: 10.1186/s12889-024-18128-2 (PMC10895720; doi:10.1186/s12889-024-18128-2)
Supplement: Supplementary file 1 — Supplementary Material 1. [file 12889_2024_18128_MOESM1_ESM.doc]

**Supplementary material**

**Supplementary Table 1** Distribution of the pulmonary function in NHANES 2007–2012.

**Supplementary Table 2** Association between sedentary behavior and pulmonary function in the NHANES 2007–2012.

**Supplementary Table 3** Joint association between sedentary behavior, MVPA time and pulmonary function stratified by sex in the NHANES 2007–2012.

**Supplementary Table 4** Joint association between sedentary behavior, MVPA time and pulmonary function stratified by age in the NHANES 2007–2012.

**Supplementary Table 5** Joint association of sedentary behavior, MVPA time and pulmonary function restricted to individuals without lung disease in the NHANES 2007–2012.

**Supplementary Table 6** Joint association of sedentary behavior, MVPA time and pulmonary function in individuals filling in the missing values in the NHANES 2007–2012.

**Supplementary Figure 1** Joint association between sedentary behavior, MVPA time and pulmonary function in males in the NHANES 2007–2012.

The model was adjusted for age, family income to poverty ratio, height, body mass index, race, serum cotinine, lung diseases, and NHANES cycles

FEV_1_: forced expiratory volume in 1 second; FVC: forced vital capacity; PEF: peak expiratory flow; FEF_25-75%_: forced expiratory flow between 25% and 75% of vital capacity; MVPA: moderate or vigorous physical activity.

**Supplementary Figure 2** Joint association between sedentary behavior, MVPA time and pulmonary function in females in the NHANES 2007–2012.

The model was adjusted for age, family income to poverty ratio, height, body mass index, race, serum cotinine, lung diseases, and NHANES cycles

FEV_1_: forced expiratory volume in 1 second; FVC: forced vital capacity; PEF: peak expiratory flow; FEF_25-75%_: forced expiratory flow between 25% and 75% of vital capacity; MVPA: moderate or vigorous physical activity.

**Supplementary Figure 3** Joint association between sedentary behavior, MVPA time and pulmonary function in children aged 12-19 years in the NHANES 2007–2012.

The model was adjusted for sex, age, family income to poverty ratio, height, body mass index, race, serum cotinine, lung diseases, and NHANES cycles

FEV_1_: forced expiratory volume in 1 second; FVC: forced vital capacity; PEF: peak expiratory flow; FEF_25-75%_: forced expiratory flow between 25% and 75% of vital capacity; MVPA: moderate or vigorous physical activity.

**Supplementary Figure 4** Joint association between sedentary behavior, MVPA time and pulmonary function in adults aged 20-44 years in the NHANES 2007–2012.

The model was adjusted for sex, age, family income to poverty ratio, height, body mass index, race, serum cotinine, lung diseases, and NHANES cycles

FEV_1_: forced expiratory volume in 1 second; FVC: forced vital capacity; PEF: peak expiratory flow; FEF_25-75%_: forced expiratory flow between 25% and 75% of vital capacity; MVPA: moderate or vigorous physical activity.

**Supplementary Figure 5** Joint association between sedentary behavior, MVPA time and pulmonary function in adults aged 45-59 years in the NHANES 2007–2012.

The model was adjusted for sex, age, family income to poverty ratio, height, body mass index, race, serum cotinine, lung diseases, and NHANES cycles

FEV_1_: forced expiratory volume in 1 second; FVC: forced vital capacity; PEF: peak expiratory flow; FEF_25-75%_: forced expiratory flow between 25% and 75% of vital capacity; MVPA: moderate or vigorous physical activity.

**Supplementary Figure 6** Joint association between sedentary behavior, MVPA time and pulmonary function in adults aged ≥ 60 years in the NHANES 2007–2012.

The model was adjusted for sex, age, family income to poverty ratio, height, body mass index, race, serum cotinine, lung diseases, and NHANES cycles

FEV_1_: forced expiratory volume in 1 second; FVC: forced vital capacity; PEF: peak expiratory flow; FEF_25-75%_: forced expiratory flow between 25% and 75% of vital capacity; MVPA: moderate or vigorous physical activity.

**Supplementary Figure 7** Joint association between sedentary behavior, MVPA time and pulmonary function restricted to individuals without lung disease in the NHANES 2007–2012.

The model was adjusted for sex, age, family income to poverty ratio, height, body mass index, race, serum cotinine, and NHANES cycles

FEV_1_: forced expiratory volume in 1 second; FVC: forced vital capacity; PEF: peak expiratory flow; FEF_25-75%_: forced expiratory flow between 25% and 75% of vital capacity; MVPA: moderate or vigorous physical activity.

**Supplementary Figure 8** Joint association between sedentary behavior, MVPA time and pulmonary function in individuals filling in the missing values adults in the NHANES 2007–2012.

The model was adjusted for sex, age, family income to poverty ratio, height, body mass index, race, serum cotinine, lung diseases, and NHANES cycles

FEV_1_: forced expiratory volume in 1 second; FVC: forced vital capacity; PEF: peak expiratory flow; FEF_25-75%_: forced expiratory flow between 25% and 75% of vital capacity; MVPA: moderate or vigorous physical activity.

Supplementary Table 1 Distribution of the pulmonary function in NHANES 2007–2012.

|  | **Percentiles** | | |
| --- | --- | --- | --- |
|  | **25th (95%CI)** | **50th (95%CI)** | **75th (95%CI)** |
| **FEV_1_ (mL)** | 2637.5 (2608.6, 2666.4) | 3217.1(3191.8, 3242.4) | 3858.9 (3827.1, 3890.6) |
| **FVC (mL)** | 3358.3 (3331.4, 3385.3) | 4043.9 (4011.0, 4076.8) | 4865.5 (4824.9, 4906.2) |
| **FEV_1_:FVC** | 0.75 (0.74, 0.75) | 0.80 (0.80, 0.80) | 0.84 (0.84, 0.85) |
| **PEF (mL/ s )** | 6800.3 (6732.9, 6867.8) | 8107.6 (8016.4, 8198.8) | 9788.6 (9679.7, 9897.5) |
| **FEF_25-75_ (mL/ s)** | 2200.1(2146.0, 2254.2) | 3088.2 (3031.6, 3144.7) | 3969.4 (3909.0, 4029.7) |

FEV_1_: forced expiratory volume in 1 second; FVC: forced vital capacity; PEF: peak expiratory flow; FEF_25-75%_: forced expiratory flow between 25% and 75% of vital capacity.

Supplementary Table 2 Association between sedentary behavior and pulmonary function in the NHANES 2007–2012.

|  | **FEV_1_** | **FVC** | | | **FEV_1_: FVC** | **PEF** | **FEF_25-75%_** |
| --- | --- | --- | --- | --- | --- | --- | --- |
|  | **β (95%CI) ^a^** | | | | | | |
| **Sedentary behavior time h/day** |  | |  |  | |  |  |
| **0-4.0** | **Reference** | | **Reference** | **Reference** | | **Reference** | **Reference** |
| **4.1-8.0** | **-0.009 (-0.014, -0.004)^*^** | | **-0.011 (-0.015, -0.008)*** | 0.002 (-0.000, 0.005) | | **-0.008 (-0.013, -0.003)*** | -0.008 (-0.018, 0.002) |
| **8.1-12.0** | **-0.015 (-0.020, -0.009)^*^** | | **-0.015 (-0.019, -0.010)*** | -0.000 (-0.003, 0.003) | | **-0.013 (-0.019, -0.007)*** | **-0.018 (-0.031, -0.005)*** |
| **>12.0** | **-0.015 (-0.026, -0.004)^*^** | | **-0.016 (-0.026, -0.006)*** | 0.001 (-0.004, 0.006) | | **-0.016 (-0.025, -0.007)*** | -0.018 (-0.042, 0.007) |
| **P for trend** | < 0.001 | | < 0.001 | 0.847 | | < 0.001 | 0.011 |

FEV_1_, FVC, FEV_1_:FVC, PEF, and FEF_25-75%_ were log_10_-transformed.

^a^Adjusted for sex, age, family income to poverty ratio, height, BMI (body mass index), race, serum cotinine, lung diseases, and NHANES cycles

*Means P < 0.05

FEV_1_: forced expiratory volume in 1 second; FVC: forced vital capacity; PEF: peak expiratory flow; FEF_25-75%_: forced expiratory flow between 25% and 75% of vital capacity

Supplementary Table 3 Joint association between sedentary behavior, MVPA time and pulmonary function stratified by sex in the NHANES 2007–2012

|  | **FEV_1_** | | | **FVC** | | | | **FEV_1_: FVC** | | **PEF** | | **FEF_25-75%_** | | |
| --- | --- | --- | --- | --- | --- | --- | --- | --- | --- | --- | --- | --- | --- | --- |
|  | **Male** | | **Female** | **Male** | | | **Female** | **Male** | **Female** | **Male** | **Female** | **Male** | | **Female** |
|  | **β (95%CI) ^a^** | | | | | | | | | | | | | |
| **>12 h/day SB and 0 min/wk MVPA** | **Reference** |  | | | **Reference** |  | | **Reference** |  | **Reference** |  | | **Reference** |  |
| **>12 h/day SB and 1-149 min/wk MVPA** | 0.018  (-0.010, 0.047) | 0.008  (-0.029, 0.044) | | | 0.007  (-0.023, 0.036) | 0.020  (-0.012, 0.052) | | 0.011  (-0.007, 0.030) | -0.012  (-0.029, 0.004) | 0.026  (-0.010, 0.062) | 0.031  (-0.010, 0.073) | | 0.062  (-0.009, 0.134) | -0.021  (-0.107, 0.064) |
| **>12 h/day SB and 150-299 min/wk MVPA** | 0.025  (-0.008, 0.057) | **0.043**  **(0.012, 0.074)*** | | | 0.012  (-0.019, 0.043) | 0.044  (0.014, 0.074) | | 0.012  (-0.003, 0.028) | 0.000  (-0.014, 0.014) | 0.031  (-0.008, 0.069) | **0.040**  **(0.009, 0.071)*** | | 0.059  (-0.002, 0.120) | 0.042  (-0.032, 0.116) |
| **>12 h/day SB and** ≥ **300 min/wk MVPA** | 0.028  (-0.004, 0.060) | 0.004  (-0.035, 0.043) | | | 0.025  (-0.003, 0.054) | 0.001  (-0.036, 0.038) | | 0.002  (-0.014, 0.018) | 0.003  (-0.015, 0.021) | **0.036**  **(0.006, 0.066)*** | 0.024  (-0.017, 0.064) | | 0.037  (-0.036, 0.110) | 0.016  (-0.063, 0.095) |
| **8.1-12 h/day SB and 0 min/wk MVPA** | 0.002  (-0.021, 0.024) | 0.010  (-0.016, 0.036) | | | 0.001  (-0.020, 0.021) | 0.015  (-0.008, 0.038) | | 0.001  (-0.015, 0.017) | -0.005  (-0.014, 0.004) | 0.011  (-0.014, 0.037) | 0.019  (-0.010, 0.048) | | 0.015  (-0.043, 0.074) | -0.003  (-0.051, 0.045) |
| **8.1-12 h/day SB and 1-149 min/wk MVPA** | 0.020  (-0.011, 0.052) | 0.016  (-0.009, 0.042) | | | 0.015  (-0.012, 0.042) | 0.018  (-0.005, 0.041) | | 0.005  (-0.010, 0.020) | -0.002  (-0.011, 0.008) | 0.025  (-0.002, 0.052) | **0.033**  **(0.004, 0.062)*** | | 0.039  (-0.030, 0.107) | 0.021  (-0.030, 0.072) |
| **8.1-12 h/day SB and 150-299 min/wk MVPA** | 0.020  (-0.007, 0.047) | 0.015  (-0.009, 0.038) | | | 0.012  (-0.010, 0.034) | 0.022  (-0.000, 0.045) | | 0.008  (-0.007, 0.022) | -0.008  (-0.019, 0.004) | **0.030**  **(0.003, 0.056)*** | 0.021  (-0.002, 0.045) | | 0.040  (-0.023, 0.104) | 0.001  (-0.050, 0.052) |
| **8.1-12 h/day SB and** ≥ **300 min/wk MVPA** | 0.016  (-0.006, 0.038) | 0.016  (-0.007, 0.040) | | | 0.012  (-0.008, 0.031) | 0.019  (-0.004, 0.042) | | 0.004  (-0.009, 0.018) | -0.003  (-0.011, 0.006) | 0.020  (-0.004, 0.044) | **0.029**  **(0.002, 0.055)*** | | 0.032  (-0.024, 0.087) | 0.018  (-0.028, 0.063) |
| **4.1-8 h/day SB and 0 min/wk MVPA** | 0.015  (-0.010, 0.039) | 0.014  (-0.012, 0.040) | | | 0.007  (-0.015, 0.029) | 0.018  (-0.006, 0.041) | | 0.007  (-0.005, 0.020) | -0.004  (-0.013, 0.006) | 0.020  (-0.002, 0.042) | 0.023  (-0.004, 0.051) | | 0.039  (-0.016, 0.094) | 0.011  (-0.041, 0.063) |
| **4.1-8 h/day SB and 1-149 min/wk MVPA** | 0.022  (-0.004, 0.048) | 0.026  (-0.001, 0.052) | | | 0.013  (-0.009, 0.035) | **0.027**  **(0.002, 0.052)*** | | 0.009  (-0.006, 0.023) | -0.002  (-0.010, 0.007) | 0.021  (-0.003, 0.044) | **0.036**  **(0.010, 0.062)*** | | 0.057  (-0.003, 0.117) | 0.024  (-0.026, 0.074) |
| **4.1-8 h/day SB and 150-299 min/wk MVPA** | 0.020  (-0.004, 0.044) | 0.018  (-0.010, 0.045) | | | 0.015  (-0.006, 0.036) | 0.023  (-0.000, 0.047) | | 0.005  (-0.008, 0.018) | -0.005  (-0.016, 0.006) | **0.033**  **(0.008, 0.058)*** | **0.033**  **(0.003, 0.063)*** | | 0.039  (-0.017, 0.094) | 0.011  (-0.045, 0.066) |
| **4.1-8 h/day SB and** ≥ **300 min/wk MVPA** | **0.024**  **(0.001, 0.048)*** | 0.016  (-0.008, 0.041) | | | 0.018  (-0.003, 0.038) | 0.020  (-0.002, 0.042) | | 0.007  (-0.007, 0.021) | -0.003  (-0.012, 0.006) | **0.027**  **(0.006, 0.049)*** | **0.030**  **(0.005, 0.056)*** | | 0.044  (-0.013, 0.101) | 0.010  (-0.038, 0.058) |
| **0-4 h/day SB and 0 min/wk MVPA** | **0.027**  **(0.003, 0.051)*** | 0.022  (-0.001, 0.045) | | | **0.024**  **(0.004, 0.044)*** | **0.026**  **(0.004, 0.049)*** | | 0.003  (-0.010, 0.016) | -0.004  (-0.013, 0.005) | **0.031**  **(0.009, 0.053)*** | **0.031**  **(0.007, 0.055)*** | | 0.045  (-0.012, 0.102) | 0.020  (-0.025, 0.065) |
| **0-4 h/day SB and 1-149 min/wk MVPA** | **0.032**  **(0.007, 0.057)*** | **0.024**  **(0.000, 0.048)*** | | | **0.027**  **(0.004, 0.050)*** | **0.031**  **(0.008, 0.055)*** | | 0.005  (-0.007, 0.018) | -0.007  (-0.016, 0.002) | **0.036**  **(0.009, 0.063)*** | **0.033**  **(0.007, 0.059)*** | | 0.053  (-0.004, 0.110) | 0.015  (-0.031, 0.060) |
| **0-4 h/day SB and 150-299 min/wk MVPA** | **0.031**  **(0.005, 0.057)*** | **0.029**  **(0.003, 0.055)*.**  **.** | | | **0.027**  **(0.003, 0.050)*** | **0.036**  **(0.012, 0.060)*** | | 0.005  (-0.011, 0.021) | -0.007  (-0.016, 0.003) | **0.039**  **(0.015, 0.064)*** | **0.034**  **(0.008, 0.061)*** | | 0.057  (-0.006, 0.121) | 0.018  (-0.034, 0.069) |
| **0-4 h/day SB and** ≥ **300 min/wk MVPA** | **0.030**  **(0.006, 0.054)*** | **0.034**  **(0.009, 0.059)*** | | | **0.025**  **(0.004, 0.045)*** | **0.037**  **(0.013, 0.060)*** | | 0.005  (-0.007, 0.018) | -0.003  (-0.012, 0.006) | **0.038**  **(0.017, 0.060)*** | **0.041**  **(0.014, 0.068)*** | | 0.051  (-0.006, 0.109) | 0.033  (-0.017, 0.082) |

FEV_1_, FVC, FEV_1_:FVC, PEF, and FEF_25-75%_ were log_10_-transformed.

^a^ Adjusted for age, family income to poverty ratio, height, BMI (body mass index), race, serum cotinine, lung diseases, and NHANES cycles

*Means P < 0.05

FEV_1_: forced expiratory volume in 1 second; FVC: forced vital capacity; PEF: peak expiratory flow; FEF_25-75%_: forced expiratory flow between 25% and 75% of vital capacity; MVPA: moderate or vigorous physical activity; SB: sedentary behavior.

Supplementary Table 4 Joint association between sedentary behavior, MVPA time and pulmonary function stratified by age in the NHANES 2007–2012 (N = 12,343)

|  | **FEV_1_** | | | | | **FVC** | | | | **FEV_1_: FVC** | | | | **PEF** | | | | **FEF_25-75%_** | | | |
| --- | --- | --- | --- | --- | --- | --- | --- | --- | --- | --- | --- | --- | --- | --- | --- | --- | --- | --- | --- | --- | --- |
|  | **12-19** | **20- 44** | | **45-59** | **≥ 60** | **12-19** | **20- 44** | **45-59** | **≥ 60** | **12-19** | **20- 44** | **45-59** | **≥ 60** | **12-19** | **20- 44** | **45-59** | **≥ 60** | **12-19** | **20- 44** | **45-59** | **≥ 60** |
|  | **β (95%CI) ^a^** | | | | | | | | | | | | | | | | | | | | |
| **>12 h/day SB and 0 min/wk MVPA** | **Reference** | | | | | **Reference** | | | | **Reference** | | | | **Reference** | | | | **Reference** | | | |
| **>12 h/day SB and 1-149 min/wk MVPA** | -0.017  (-0.057, 0.023) | | -0.006  (-0.034, 0.022) | 0.005  (-0.033, 0.043) | 0.057  (-0.003, 0.116) | 0.017  (-0.014, 0.048) | -0.009  (-0.037, 0.020) | 0.005  (-0.030, 0.039) | 0.027  (-0.023, 0.078) | -0.034  (-0.056, 0.013) | 0.003  (-0.011, 0.016) | 0.001  (-0.024, 0.025) | 0.029  (-0.006, 0.052) | -0.027  (-0.072, 0.018) | -0.002  (-0.032, 0.029) | **0.052**  **(0.008, 0.097)*** | 0.127  (-0.032, 0.223) | -0.087  (-0.169, 0.006) | 0.001  (-0.052, 0.054) | -0.005  (-0.113, 0.103) | 0.134  (-0.005, 0.262) |
| **>12 h/day SB and 150-299 min/wk MVPA** | -0.014  (-0.046, 0.019) | | 0.016  (-0.019, 0.051) | 0.001  (-0.042, 0.043) | 0.095  (-0.032, 0.157) | 0.004  (-0.029, 0.037) | 0.012  (-0.025, 0.050) | 0.000  (-0.040, 0.040) | 0.055  (-0.002, 0.113) | -0.018  (-0.036, 0.001) | 0.004  (-0.009, 0.017) | 0.001  (-0.022, 0.025) | 0.039  (-0.011, 0.068) | -0.028  (-0.079, 0.024) | 0.023  (-0.018, 0.063) | 0.019  (-0.028, 0.066) | 0.063  (-0.003, 0.123) | -0.067  (-0.114, 0.021) | 0.015  (-0.039, 0.068) | -0.009  (-0.133, 0.116) | 0.235  (-0.119, 0.351) |
| **>12 h/day SB and** ≥ **300 min/wk MVPA** | -0.011  (-0.043, 0.022) | | 0.002  (-0.021, 0.026) | **0.046**  **(0.004, 0.087)*** | 0.047  (-0.035, 0.129) | 0.008  (-0.030, 0.045) | 0.012  (-0.012, 0.036) | 0.030  (-0.004, 0.063) | 0.026  (-0.035, 0.087) | -0.018  (-0.034, 0.002) | -0.010  (-0.026, 0.007) | 0.016  (-0.006, 0.038) | 0.021  (-0.022, 0.064) | 0.003  (-0.041, 0.047) | 0.023  (-0.004, 0.050) | **0.058**  **(0.015, 0.101)*** | 0.039  (-0.047, 0.126) | -0.046  (-0.094, 0.002) | -0.026  (-0.079, 0.026) | 0.083  (-0.019, 0.186) | 0.123  (-0.073, 0.320) |
| **8.1-12 h/day SB and 0 min/wk MVPA** | -0.015  (-0.042, 0.011) | | -0.001  (-0.021, 0.018) | 0.018  (-0.013, 0.050) | 0.010  (-0.044, 0.065) | 0.003  (-0.022, 0.027) | 0.001  (-0.020, 0.021) | 0.018  (-0.007, 0.044) | 0.003  (-0.046, 0.052) | -0.018  (-0.028, 0.008) | -0.002  (-0.014, 0.010) | 0.000  (-0.021, 0.021) | 0.008  (-0.013, 0.028) | -0.008  (-0.046, 0.030) | 0.009  (-0.012, 0.030) | **0.032**  **(0.001, 0.063)*** | 0.014  (-0.044, 0.071) | -0.053  (-0.094, 0.011) | -0.014  (-0.055, 0.028) | 0.020  (-0.067, 0.106) | 0.073  (-0.016, 0.161) |
| **8.1-12 h/day SB and 1-149 min/wk MVPA** | -0.009  (-0.041, 0.023) | | -0.007  (-0.028, 0.014) | 0.031  (-0.004, 0.059) | 0.038  (-0.031, 0.106) | 0.014  (-0.015, 0.042) | -0.003  (-0.026, 0.021) | 0.021  (-0.003, 0.045) | 0.008  (-0.049, 0.064) | -0.023  (-0.034, 0.011) | -0.004  (-0.017, 0.008) | 0.010  (-0.008, 0.029) | 0.030  (-0.009, 0.051) | 0.003  (-0.042, 0.048) | 0.012  (-0.010, 0.034) | **0.040**  **(0.012, 0.068)*** | 0.032  (-0.032, 0.097) | -0.048  (-0.100, 0.004) | -0.028  (-0.067, 0.012) | 0.055  (-0.025, 0.135) | **0.161**  **(0.040, 0.282)*** |
| **8.1-12 h/day SB and 150-299 min/wk MVPA** | -0.004  (-0.033, 0.025) | | 0.003  (-0.019, 0.025) | 0.029  (-0.008, 0.066) | 0.026  (-0.039, 0.090) | 0.011  (-0.013, 0.036) | 0.007  (-0.016, 0.030) | 0.026  (-0.002, 0.054) | 0.011  (-0.049, 0.070) | -0.015  (-0.025, 0.006) | -0.004  (-0.017, 0.009) | 0.003  (-0.020, 0.026) | 0.015  (-0.007, 0.036) | -0.003  (-0.046, 0.041) | 0.008  (-0.012, 0.029) | **0.058**  **(0.028, 0.089)*** | 0.024  (-0.038, 0.086) | -0.027  (-0.073, 0.019) | -0.017  (-0.063, 0.028) | 0.039  (-0.070, 0.149) | 0.091  (-0.012, 0.193) |
| **8.1-12 h/day SB and** ≥ **300 min/wk MVPA** | -0.008  (-0.031, 0.015) | | 0.009  (-0.007, 0.025) | **0.045**  **(0.014, 0.076)*** | 0.054  (-0.016, 0.124) | 0.010  (-0.013, 0.033) | 0.014  (-0.003, 0.030) | **0.035**  **(0.009, 0.062)*** | 0.028  (-0.030, 0.087) | -0.018  (-0.026, 0.011) | -0.005  (-0.017, 0.007) | 0.010  (-0.009, 0.029) | 0.026  (-0.003, 0.048) | 0.003  (-0.032, 0.038) | 0.015  (-0.005, 0.035) | **0.060**  **(0.033, 0.087)*** | 0.071  (-0.003, 0.138) | -0.039  (-0.072, 0.005) | -0.007  (-0.046, 0.033) | 0.077  (-0.009, 0.162) | **0.153**  **(0.039, 0.267)*** |
| **4.1-8 h/day SB and 0 min/wk MVPA** | -0.009  (-0.039, 0.021) | | 0.000  (-0.017, 0.017) | 0.031  (-0.001, 0.062) | 0.015  (-0.043, 0.073) | 0.011  (-0.017, 0.038) | 0.001  (-0.017, 0.018) | 0.019  (-0.005, 0.044) | 0.005  (-0.044, 0.055) | -0.020  (-0.029, 0.011) | 0.000  (-0.012, 0.012) | 0.012  (-0.005, 0.029) | 0.010  (-0.010, 0.029) | -0.006  (-0.043, 0.031) | 0.005  (-0.012, 0.022) | **0.046**  **(0.019, 0.073)*** | 0.021  (-0.037, 0.078) | -0.048  (-0.093, 0.003) | -0.009  (-0.046, 0.029) | 0.065  (-0.018, 0.147) | **0.076**  **(0.018, 0.170)*** |
| **4.1-8 h/day SB and 1-149 min/wk MVPA** | -0.002  (-0.028, 0.024) | | 0.005  (-0.016, 0.025**)** | 0.029  (-0.003, 0.062) | 0.039  (-0.021, 0.100) | 0.018  (-0.008, 0.043) | 0.005  (-0.018, 0.027) | 0.021  (-0.005, 0.046) | 0.017  (-0.035, 0.069) | **-**0.019  (-0.028, 0.011) | 0.000  (-0.012, 0.012) | 0.009  (-0.009, 0.027) | 0.022  (-0.001, 0.045) | -0.001  (-0.039, 0.036) | 0.007  (-0.013, 0.027) | **0.044**  **(0.017, 0.071)*** | 0.048  (-0.013, 0.109) | -0.043  (-0.077, 0.009) | -0.007  (-0.044, 0.030) | 0.060  (-0.024, 0.144) | **0.135**  **(0.030, 0.241)*** |
| **4.1-8 h/day SB and 150-299 min/wk MVPA** | -0.007  (-0.036, 0.022) | | 0.001  (-0.020, 0.021) | 0.017  (-0.019, 0.052) | 0.039  (-0.021, 0.099) | 0.008 (-0.017, 0.033) | 0.005 (-0.015, 0.026) | 0.012 (-0.017, 0.041) | 0.020 (-0.031, 0.071) | -0.015 (-0.028, 0.003) | -0.005 (-0.015, 0.006) | 0.005 (-0.014, 0.024) | 0.019 (-0.001, 0.039) | 0.001 (-0.040, 0.041) | 0.019 (-0.004, 0.041) | **0.034 (0.001, 0.066)*** | 0.046 (-0.016, 0.107) | -0.038 (-0.088, 0.013) | -0.016 (-0.056, 0.025) | 0.030 (-0.055, 0.116) | **0.122 (0.024, 0.220)*** |
| **4.1-8 h/day SB and** ≥ **300 min/wk MVPA** | -0.005  (-0.029, 0.019) | | 0.007  (-0.011, 0.024) | **0.044**  **(0.014, 0.073)*** | 0.033 (-0.032, 0.097) | 0.014  (-0.009, 0.038) | 0.007  (-0.011, 0.026) | **0.032**  **(0.008, 0.056)*** | 0.022  (-0.030, 0.074) | -0.019  (-0.028, 0.010) | 0.000  (-0.013, 0.013) | 0.012  (-0.005, 0.029) | 0.011  (-0.013, 0.034) | 0.004  (-0.030, 0.039) | 0.017  (-0.003, 0.036) | **0.055**  **(0.029, 0.082)*** | 0.042  (-0.021, 0.105) | -0.042  (-0.078, 0.006) | -0.002  (-0.040, 0.037) | 0.071  (-0.007, 0.149) | **0.092**  **(0.023, 0.206)*** |
| **0-4 h/day SB and 0 min/wk MVPA** | -0.014  (-0.043, 0.015) | | 0.000  (-0.019, 0.019) | 0.027  (-0.002, 0.055) | 0.038  (-0.017, 0.092) | 0.009  (-0.017, 0.036) | 0.005  (-0.014, 0.024) | 0.024  (-0.000, 0.048) | 0.018  (-0.027, 0.064) | -0.023  (-0.039, 0.008) | -0.005  (-0.017, 0.006) | 0.003  (-0.014, 0.020) | 0.019  (-0.000, 0.038) | -0.011  (-0.053, 0.032) | 0.006  (-0.011, 0.023) | **0.036**  **(0.011, 0.062)*** | 0.041  (-0.014, 0.095) | -0.054  (-0.108, 0.001) | -0.019  (-0.056, 0.018) | 0.036  (-0.042, 0.114) | **0.121**  **(0.031, 0.211)*** |
| **0-4 h/day SB and 1-149 min/wk MVPA** | -0.011  (-0.037, 0.015) | | 0.000  (-0.016, 0.016) | **0.043**  **(0.012, 0.075)*** | 0.026 (-0.031, 0.083) | 0.006 (-0.020, 0.032) | 0.007 (-0.011, 0.025) | **0.037 (0.010, 0.065)*** | 0.010 (-0.038, 0.058) | -0.017 (-0.032, 0.002) | -0.007 (-0.020, 0.006) | 0.006 (-0.012, 0.024) | 0.016 (-0.005, 0.036) | 0.002 (-0.035, 0.039) | 0.009 (-0.008, 0.027) | **0.049 (0.018, 0.080)*** | 0.024 (-0.038, 0.086) | -0.036 (-0.088, 0.016) | -0.025 (-0.064, 0.015) | 0.052 (-0.029, 0.133) | **0.104 (0.007, 0.200)*** |
| **0-4 h/day SB and 150-299 min/wk MVPA** | -0.013  (-0.039, 0.013) | | 0.005  (-0.016, 0.025) | **0.036**  **(0.002, 0.070)*** | 0.036  (-0.026, 0.097) | 0.014  (-0.016, 0.044) | 0.006  (-0.015, 0.027) | **0.036**  **(0.011, 0.061)*** | 0.018  (-0.033, 0.069) | -0.027  (-0.047, 0.007) | -0.001  (-0.013, 0.011) | 0.000  (-0.023, 0.024) | 0.018  (-0.002, 0.037) | -0.009  (-0.051, 0.033) | 0.012  (-0.007, 0.032) | **0.044**  **(0.008, 0.079)*** | 0.040  (-0.021, 0.101) | -0.067  (-0.122, 0.012) | -0.005  (-0.043, 0.033) | 0.045  (-0.050, 0.140) | **0.109**  **(0.007, 0.212)*** |
| **0-4 h/day SB and** ≥ **300 min/wk MVPA** | -0.008  (-0.036, 0.019) | | 0.003  (-0.015 0.021) | **0.042**  **(0.011, 0.073)*** | 0.044  (-0.016, 0.105) | 0.012  (-0.014, 0.038) | 0.006  (-0.012, 0.024) | **0.034**  **(0.009, 0.059)*** | 0.029  (-0.023, 0.080) | -0.020  (-0.029, 0.012) | -0.003  (-0.015, 0.008) | 0.008  (-0.010, 0.025) | 0.016  (-0.005, 0.036) | 0.000  (-0.038, 0.038) | 0.013  (-0.006, 0.032) | **0.051**  **(0.020, 0.082)*** | 0.044  (-0.015, 0.103) | -0.046  (-0.082, 0.010) | -0.012  (-0.052, 0.028) | 0.057  (-0.024, 0.138) | **0.119**  **(0.017, 0.222)*** |

FEV_1_, FVC, FEV_1_:FVC, PEF, and FEF_25-75%_ were log_10_-transformed.

^a^ Adjusted for sex, age, family income to poverty ratio, height, BMI (body mass index), race, serum cotinine, lung diseases, and NHANES cycles

*Means P < 0.05

FEV_1_: forced expiratory volume in 1 second; FVC: forced vital capacity; PEF: peak expiratory flow; FEF_25-75%_: forced expiratory flow between 25% and 75% of vital capacity; MVPA: moderate or vigorous physical activity; SB: sedentary behavior.

Supplementary Table 5 Joint association of sedentary behavior, MVPA time and pulmonary function restricted to individuals without lung disease in the NHANES 2007–2012.

|  | **FEV_1_** | **FVC** | | | **FEV_1_: FVC** | **PEF** | **FEF_25-75%_** |
| --- | --- | --- | --- | --- | --- | --- | --- |
|  | **β (95%CI) ^a^** | | | | | | |
| **>12 h/day SB and 0 min/wk MVPA** | **Reference** | | **Reference** | **Reference** | | **Reference** | **Reference** |
| **>12 h/day SB and 1-149 min/wk MVPA** | 0.006 (-0.020, 0.033) | | 0.001 (-0.017, 0.036) | -0.003 (-0.016, 0.009) | | 0.015 (-0.016, 0.046) | -0.007 (-0.062, 0.048) |
| **>12 h/day SB and 150-299 min/wk MVPA** | 0.026 (-0.002, 0.054) | | 0.023 (-0.008, 0.053) | 0.004 (-0.008, 0.015) | | 0.026 (-0.007, 0.060) | 0.033 (-0.015, 0.081) |
| **>12 h/day SB and** ≥ **300 min/wk MVPA** | 0.012 (-0.013, 0.037) | | 0.016 (-0.009, 0.041) | -0.004 (-0.017, 0.009) | | **0.030 (0.001, 0.059)*** | -0.004 (-0.058, 0.050) |
| **8.1-12 h/day SB and 0 min/wk MVPA** | 0.004 (-0.017, 0.024) | | 0.007 (-0.015, 0.028) | -0.003 (-0.013, 0.007) | | 0.016 (-0.005, 0.037) | -0.010 (-0.052, 0.033) |
| **8.1-12 h/day SB and 1-149 min/wk MVPA** | 0.008 (-0.015, 0.031) | | 0.006 (-0.017, 0.030) | 0.002 (-0.009, 0.013) | | **0.022 (0.001, 0.043)*** | 0.010 (-0.035, 0.055) |
| **8.1-12 h/day SB and 150-299 min/wk MVPA** | 0.011 (-0.011, 0.032) | | 0.013 (-0.009, 0.035) | -0.002 (-0.013, 0.009) | | 0.019 (-0.001, 0.039) | -0.002 (-0.049, 0.044) |
| **8.1-12 h/day SB and** ≥ **300 min/wk MVPA** | 0.013 (-0.008, 0.033) | | 0.012 (-0.009, 0.033) | 0.000 (-0.010, 0.011) | | **0.024 (0.004, 0.043)*** | 0.010 (-0.033, 0.053) |
| **4.1-8 h/day SB and 0 min/wk MVPA** | 0.010 (-0.011, 0.032) | | 0.010 (-0.011, 0.030) | 0.001 (-0.009, 0.010) | | 0.020 (-0.000, 0.040) | 0.008 (-0.035, 0.052) |
| **4.1-8 h/day SB and 1-149 min/wk MVPA** | 0.021 (-0.001, 0.043) | | 0.016 (-0.006, 0.038) | 0.005 (-0.005, 0.015) | | **0.031 (0.010, 0.051)*** | 0.031 (-0.013, 0.075) |
| **4.1-8 h/day SB and 150-299 min/wk MVPA** | 0.014 (-0.007, 0.035) | | 0.014 (-0.007, 0.034) | 0.000 (-0.010, 0.010) | | **0.031 (0.011, 0.051)*** | 0.010 (-0.035, 0.054) |
| **4.1-8 h/day SB and** ≥ **300 min/wk MVPA** | 0.017 (-0.004, 0.037) | | 0.015 (-0.006, 0.035) | 0.002 (-0.008, 0.012) | | **0.028 (0.009, 0.047)*** | 0.014 (-0.027, 0.054) |
| **0-4 h/day SB and 0 min/wk MVPA** | 0.017 (-0.004, 0.037) | | 0.018 (-0.003, 0.039) | -0.001 (-0.011, 0.008) | | **0.029 (0.010, 0.049)*** | 0.014 (-0.028, 0.056) |
| **0-4 h/day SB and 1-149 min/wk MVPA** | 0.019 (-0.003, 0.041) | | **0.022 (0.001, 0.044)*** | -0.003 (-0.013, 0.007) | | **0.029 (0.007, 0.050)*** | 0.009 (-0.037, 0.054) |
| **0-4 h/day SB and 150-299 min/wk MVPA** | **0.024 (0.002, 0.046)*** | | **0.025 (0.003, 0.046)*** | 0.000 (-0.011, 0.011) | | **0.034 (0.012, 0.055)*** | 0.024 (-0.024, 0.071) |
| **0-4 h/day SB and** ≥ **300 min/wk MVPA** | **0.026 (0.005, 0.046)*** | | **0.024 (0.004, 0.044)*** | 0.002 (-0.008, 0.011) | | **0.037 (0.016, 0.058)*** | 0.028 (-0.015, 0.071) |

FEV_1_, FVC, FEV_1_:FVC, PEF, and FEF_25-75%_ were log_10_-transformed.

^a^ Adjusted for sex, age, family income to poverty ratio, height, BMI (body mass index), race, serum cotinine, and NHANES cycles

*Means P < 0.05

FEV_1_: forced expiratory volume in 1 second; FVC: forced vital capacity; PEF: peak expiratory flow; FEF_25-75%_: forced expiratory flow between 25% and 75% of vital capacity, MVPA: moderate or vigorous physical activity; SB: sedentary behavior

Supplementary Table 6 Joint association of sedentary behavior, MVPA time and pulmonary function in individuals filling in the missing values in the NHANES 2007–2012.

|  | **FEV_1_** | **FVC** | | | **FEV_1_: FVC** | **PEF** | **FEF_25-75%_** |
| --- | --- | --- | --- | --- | --- | --- | --- |
|  | **β (95%CI) ^a^** | | | | | | |
| **>12 h/day SB and 0 min/wk MVPA** | **Reference** | | **Reference** | **Reference** | | **Reference** | **Reference** |
| **>12 h/day SB and 1-149 min/wk MVPA** | 0.018 (-0.012, 0.046) | | 0.014 (-0.012, 0.040) | 0.003 (-0.010, 0.016) | | 0.026 (-0.009, 0.061) | 0.028 (-0.031, 0.086) |
| **>12 h/day SB and 150-299 min/wk MVPA** | **0.040 (0.019, 0.060)*** | | **0.029 (0.009, 0.049)*** | 0.011 (-0.000, 0.022) | | **0.038 (0.014, 0.063)*** | **0.068 (0.019, 0.117)*** |
| **>12 h/day SB and** ≥ **300 min/wk MVPA** | **0.030 (0.002, 0.057)*** | | **0.025 (0.003, 0.047)*** | 0.005 (-0.009, 0.018) | | **0.039 (0.011, 0.067)*** | 0.041 (-0.019, 0.100) |
| **8.1-12 h/day SB and 0 min/wk MVPA** | 0.009 (-0.015, 0.033) | | 0.008 (-0.009, 0.025) | 0.001 (-0.012, 0.014) | | 0.014 (-0.012, 0.040) | 0.014 (-0.040, 0.069) |
| **8.1-12 h/day SB and 1-149 min/wk MVPA** | 0.024 (-0.000, 0.049) | | 0.018 (-0.002, 0.037) | 0.007 (-0.004, 0.017) | | **0.030 (0.007, 0.053)*** | 0.046 (-0.004, 0.096) |
| **8.1-12 h/day SB and 150-299 min/wk MVPA** | **0.024 (0.001, 0.048)*** | | **0.020 (0.003, 0.038)*** | 0.004 (-0.008, 0.016) | | **0.028 (0.004, 0.052)*** | 0.038 (-0.015, 0.091) |
| **8.1-12 h/day SB and** ≥ **300 min/wk MVPA** | **0.021 (0.001, 0.042)*** | | **0.016 (0.001, 0.031)*** | 0.005 (-0.006, 0.016) | | **0.025 (0.004, 0.047)*** | 0.039 (-0.009, 0.087) |
| **4.1-8 h/day SB and 0 min/wk MVPA** | 0.022 (-0.000, 0.044) | | 0.016 (-0.001, 0.033) | 0.006 (-0.005, 0.016) | | **0.025 (0.003, 0.047)*** | 0.041 (-0.005, 0.088) |
| **4.1-8 h/day SB and 1-149 min/wk MVPA** | **0.032 (0.009, 0.054)*** | | **0.024 (0.006, 0.041)*** | 0.008 (-0.003, 0.019) | | **0.033 (0.012, 0.054)*** | **0.058 (0.012, 0.104)*** |
| **4.1-8 h/day SB and 150-299 min/wk MVPA** | **0.027 (0.005, 0.049)*** | | **0.022 (0.005, 0.038)*** | 0.005 (-0.005, 0.015) | | **0.036 (0.014, 0.058)*** | 0.045 (-0.001, 0.091) |
| **4.1-8 h/day SB and** ≥ **300 min/wk MVPA** | **0.028 (0.007, 0.049)*** | | **0.021 (0.005, 0.038)*** | 0.007 (-0.003, 0.017) | | **0.032 (0.011, 0.053)*** | **0.046 (0.001, 0.091)*** |
| **0-4 h/day SB and 0 min/wk MVPA** | **0.031 (0.010, 0.053)*** | | **0.028 (0.012, 0.044)*** | 0.004 (-0.006, 0.013) | | **0.033 (0.012, 0.054)*** | **0.050 (0.067, 0.094)*** |
| **0-4 h/day SB and 1-149 min/wk MVPA** | **0.033 (0.012, 0.055)*** | | **0.030 (0.013, 0.047)*** | 0.003 (-0.007, 0.013) | | **0.035 (0.013, 0.058)*** | **0.049 (0.027, 0.095)*** |
| **0-4 h/day SB and 150-299 min/wk MVPA** | **0.037 (0.012, 0.061)*** | | **0.033 (0.015, 0.052)*** | 0.003 (-0.008, 0.014) | | **0.040 (0.016, 0.064)*** | **0.052 (0.000, 0.104)*** |
| **0-4 h/day SB and** ≥ **300 min/wk MVPA** | **0.038 (0.016, 0.060)*** | | **0.032 (0.017, 0.048)*** | 0.006 (-0.005, 0.017) | | **0.042 (0.019, 0.065)*** | **0.058 (0.010, 0.107)*** |

FEV_1_, FVC, FEV_1_:FVC, PEF, and FEF_25-75%_ were log_10_-transformed.

^a^ Adjusted for sex, age, family income to poverty ratio, height, BMI (body mass index), race, serum cotinine, lung diseases, and NHANES cycles

*Means P < 0.05

FEV_1_: forced expiratory volume in 1 second; FVC: forced vital capacity; PEF: peak expiratory flow; FEF_25-75%_: forced expiratory flow between 25% and 75% of vital capacity; MVPA: moderate or vigorous physical activity; SB: sedentary behavior


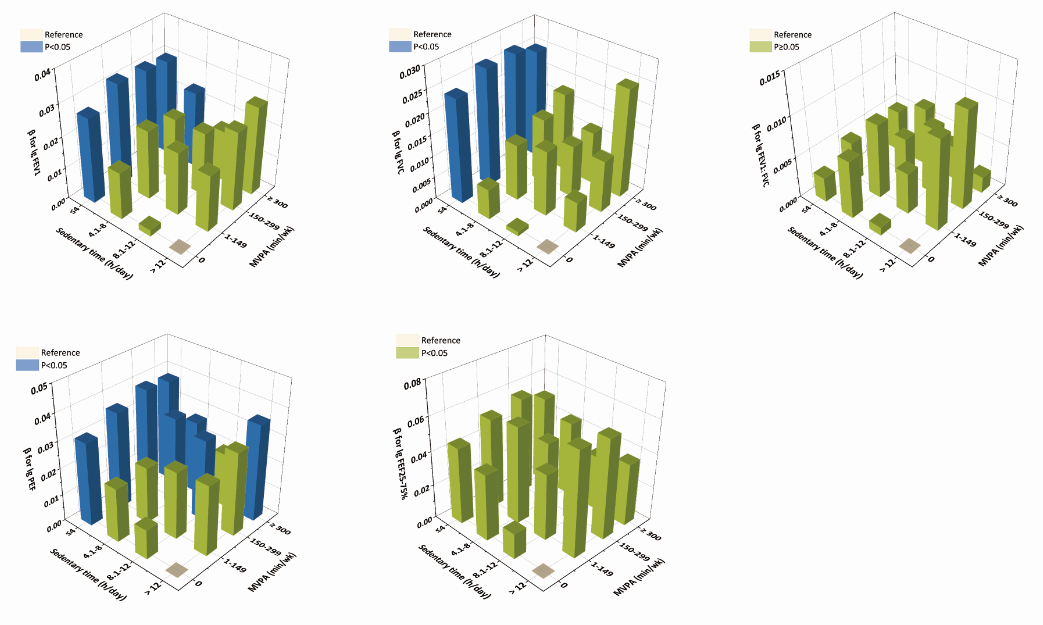


**Supplementary Figure 1**


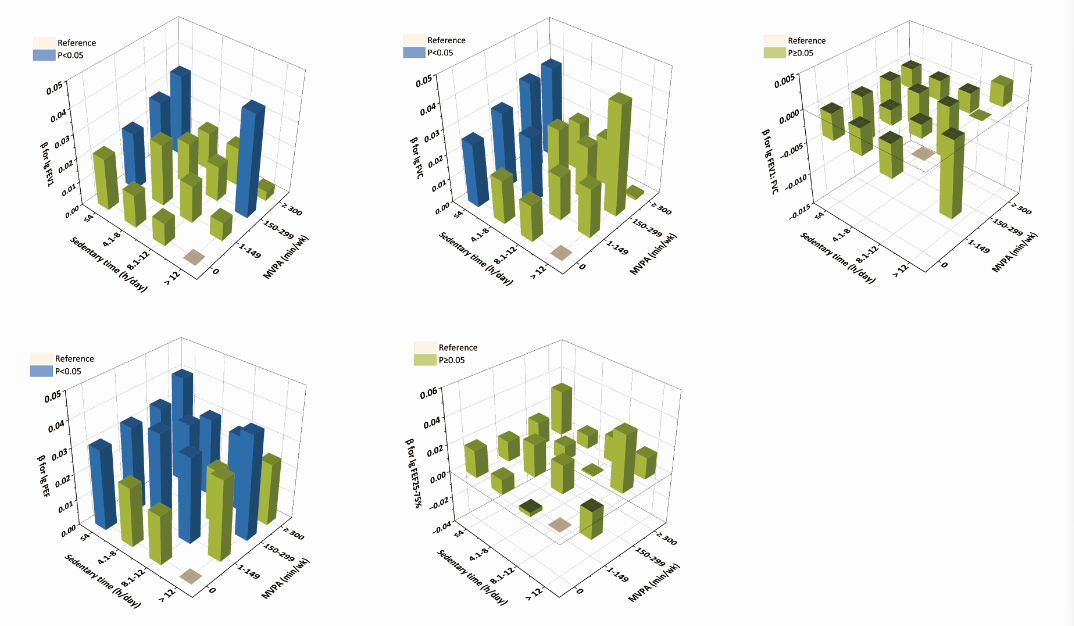


**Supplementary Figure 2**

**
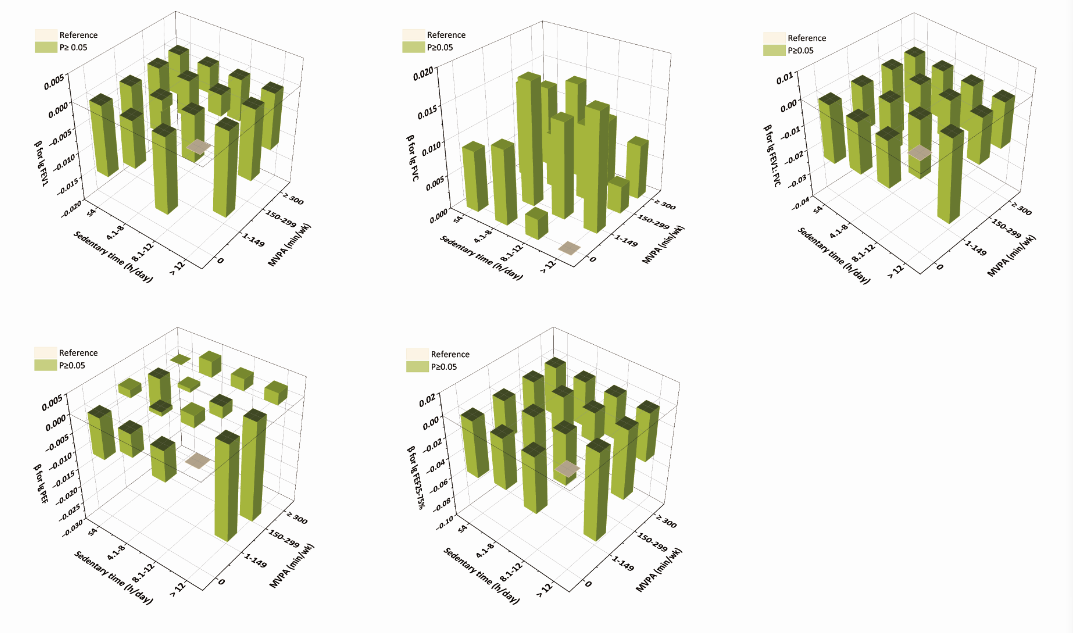
**

**Supplementary Figure 3**

**
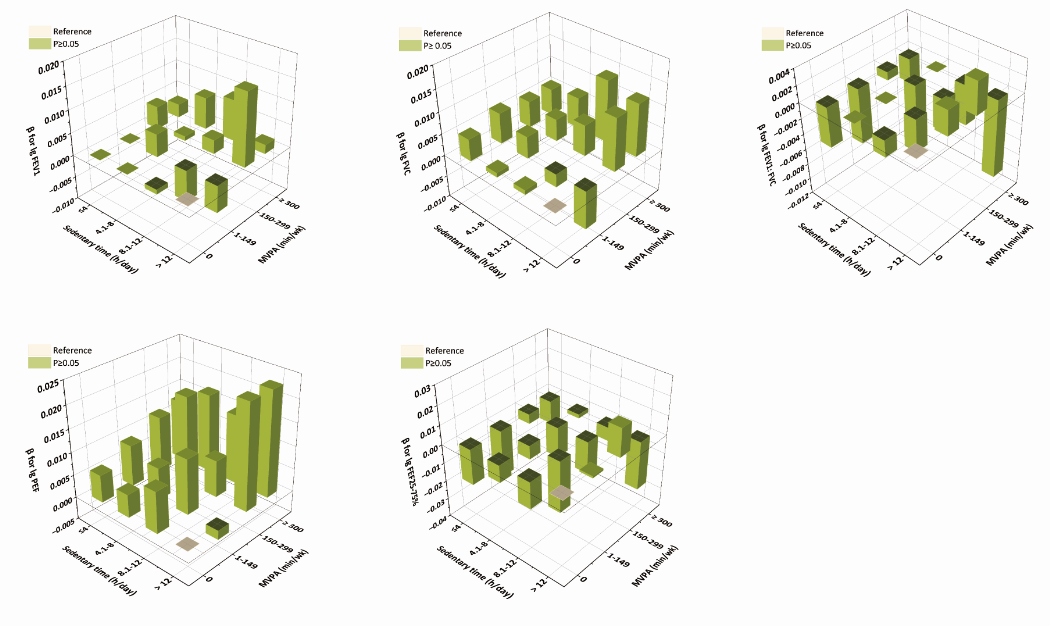
**

**Supplementary Figure 4**

**
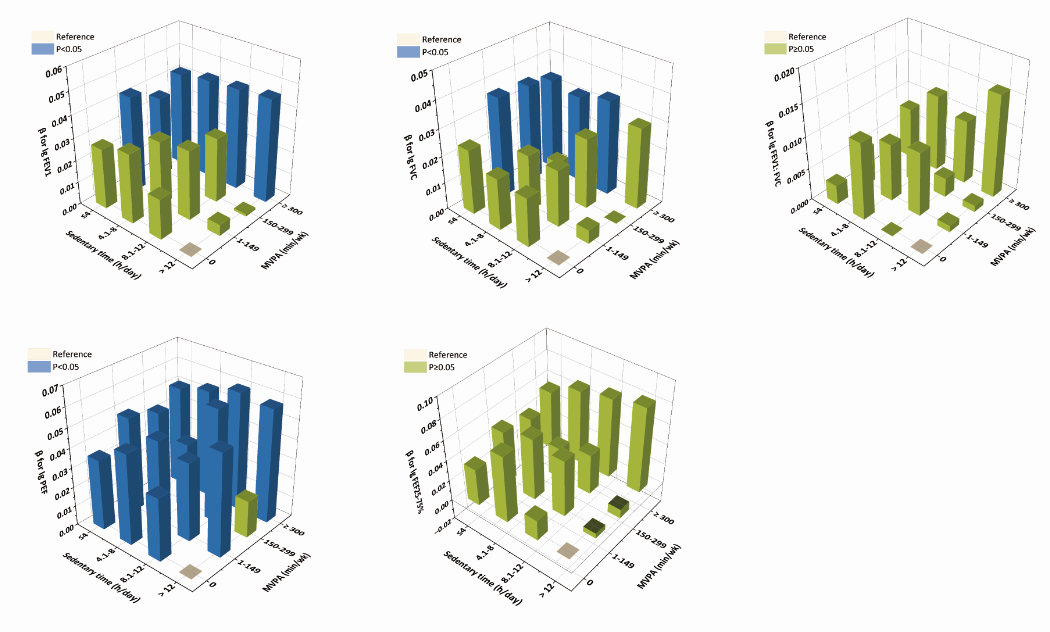
**

**Supplementary Figure 5**

**
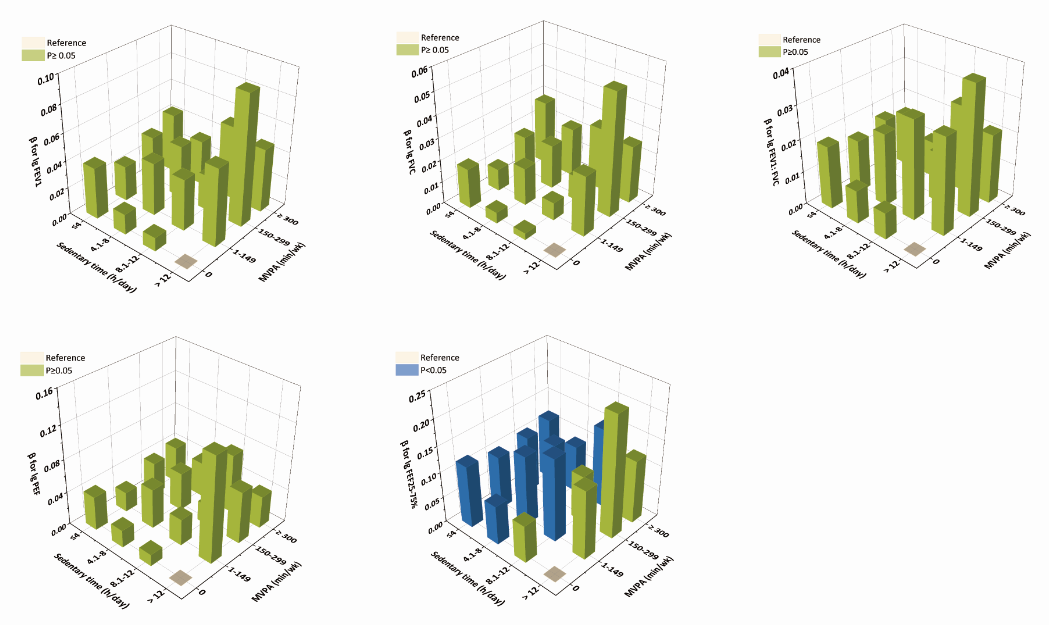
**

**Supplementary Figure 6**

**
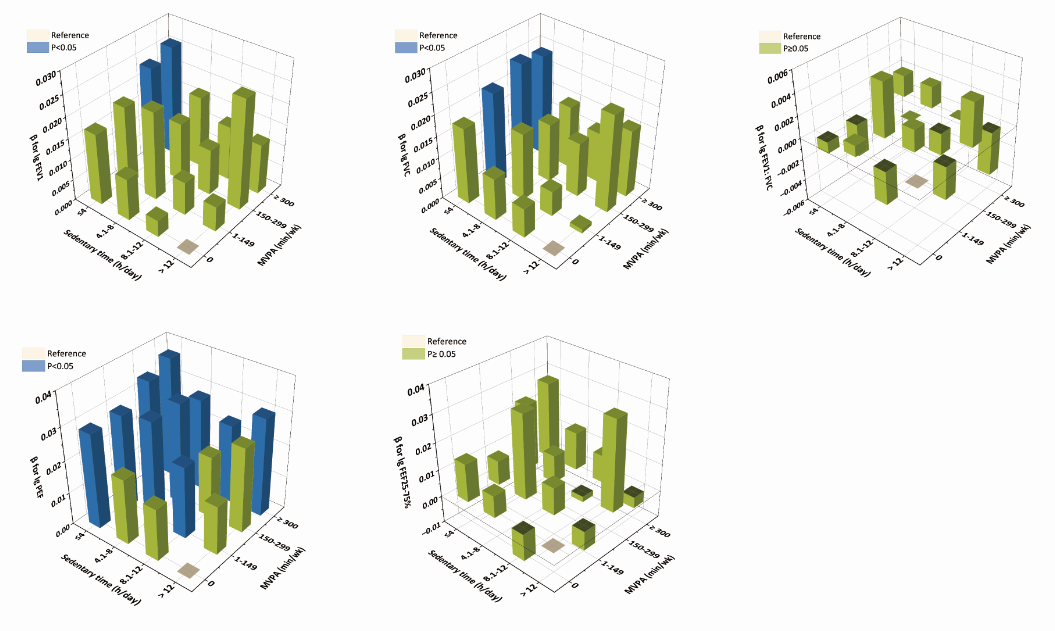
**

**Supplementary Figure 7**


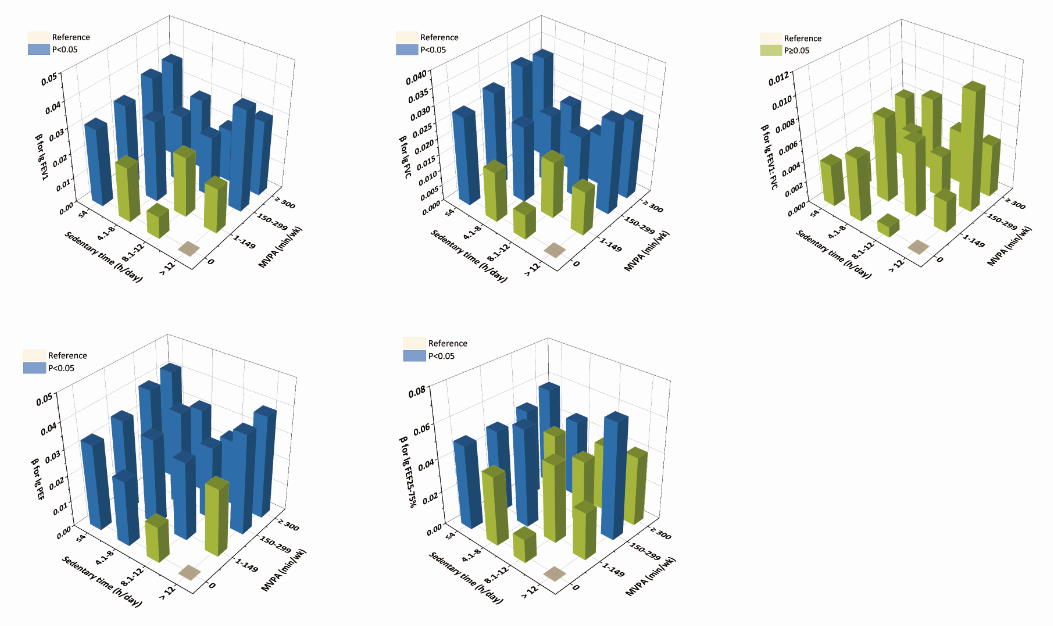


**Supplementary Figure 8**
